# Effect of Nutrition Education on Health Science University Students to Improve Cardiometabolic Profile and Inflammatory Status

**DOI:** 10.3390/nu15214685

**Published:** 2023-11-05

**Authors:** Miguel López-Moreno, Marta Garcés-Rimón, Marta Miguel-Castro, Elia Fernández-Martínez, María Teresa Iglesias López

**Affiliations:** 1 Instituto de Investigación en Ciencias de Alimentación, Consejo Superior de Investigaciones Científicas, Universidad Autónoma de Madrid, 28049 Madrid, Spain; miguel.lopez@ufv.es (M.L.-M.); marta.garces@ufv.es (M.G.-R.); marta.miguel@csic.es (M.M.-C.); 2 Grupo de Investigación en Biotecnología Alimentaria, Universidad Francisco de Vitoria, 28223 Madrid, Spain; 3 Departamento de Enfermería, Universidad de Huelva, 21007 Huelva, Spain; elia.fernandez@denf.uhu.es

**Keywords:** vitamin D, monocyte, university students, serum lipids, nutritional education

## Abstract

The inadequate lifestyle associated with university life may have a negative impact on various cardiometabolic factors. The aim of this study was to evaluate the effect of a one-year nutrition education course on cardiometabolic parameters in undergraduate health science students. During the 2021–22 academic year, 1.30 h nutrition sessions were conducted twice a week. Capillary blood samples were collected and centrifuged to measure cardiometabolic and inflammatory biomarkers in serum. The sample studied consisted of 49 students: 20.4% male and 79.6% female. The nutritional intervention resulted in changes in dietary patterns, with increased consumption of vegetables, nuts and legumes. After the course, females showed an increase in HDL-cholesterol levels (*p* = 0.007) and no change in LDL-cholesterol levels (*p* = 0.189). On the other hand, males showed significant changes in HDL-cholesterol (*p* = 0.001) and LDL-cholesterol (*p* = 0.043) levels. The atherogenic index was also significantly reduced (*p* < 0.001) in both males (*p* = 0.009) and females (*p* = 0.002). Differences were also observed in the increase in vitamin D levels in both males and females, although the magnitude of the increase was greater in the men (Δ = 7.94, *p* = 0.016 in men vs. Δ = 4.96, *p* = 0.001 in women). The monocyte-to-HDL ratio (MHR) showed a significant reduction, although these differences were only significant in males. Students with low vitamin D levels had higher LDL-cholesterol values (*p* = 0.01) and atherogenic index (*p* = 0.029). Adjusted linear regression analysis showed a significant association between post-course vitamin D MHR (β = −0.42, IC: −0.29, −0.06, *p* < 0.01). These findings suggest the importance of including nutrition education programs during the university stage for the prevention of long-term health problems.

## 1. Introduction

The global prevalence of cardiometabolic disorders among young adults is a growing concern, and failure to implement early interventions could result in substantial and long-lasting public health and economic burdens [1]. At the onset of university, students experience a significant change in lifestyle with little physical activity; an unhealthy dietary pattern including skipped meals, more frequent intake of processed and ultra-processed foods and low consumption of vegetables; as well as excessive alcohol intake [2,3,4,5,6]. In particular, students living away from their parents’ homes show a greater predisposition to poorer-quality diets, with a higher consumption of fast food [7]. In addition, the university population is a vulnerable age group because the transition to university is a critical period for these young adults with a high-perceived stress burden and the significant impact on psychological well-being [8,9]. They are, therefore, an important target population for the promotion of healthy lifestyles to reduce the risk of developing chronic non-communicable disease in adulthood [10]. The inadequate lifestyle associated with university life can have a negative impact on various cardiometabolic factors [11]. González Sandoval et al. (2014) [12] found that among Mexican university students, 61.3%, 22.6% and 32.7% had elevated LDL-cholesterol levels (>100 mg/dL), elevated total cholesterol values (200 mg/dL) and overweight/obesity, respectively. Williams et al. (2019) [13] identified that 71.0% of women and 80.9% of men in the university population met one or more metabolic syndrome factors. Body weight according to body mass index (BMI) is one of the most widely used diagnostic criteria to assess an individual’s health status and cardiovascular risk. However, a significant prevalence of individuals with a normal weight but with a high percentage of fat mass has been observed in university students, a situation known as normal-weight obesity (NWO), which is associated with an increased cardiovascular risk [14]. During COVID-19, online classes were required at universities around the world, and this new situation led to changes in food intake and lifestyle habits, including increased consumption of unhealthy diets such as sugary drinks, snacks and sweets, a decrease in physical activity and increased psychological distress in some students [15,16,17,18,19]. Vitamin D is a fat-soluble vitamin traditionally associated with calcium homeostasis and bone health, but it also has important extra-skeletal, immunological, cardiovascular and endocrine implications [20,21,22]. Recent studies have suggested that vitamin D deficiency may also be associated with increased susceptibility and worse prognosis of coronavirus disease 2019 (COVID-19) [23,24]. In the general population, the Institute of Medicine (IOM) sets the optimal 25-hydroxyvitamin D [25(OH)D] concentration in the range >20 ng/mL, while other institutions, such as the Endocrine Society, recommend values above 30 ng/mL [25,26]. Vitamin D deficiency is a common situation worldwide and represents a major public health problem. It is estimated that 40% of the European population is vitamin D deficient (<20 ng/mL) [27,28]. Even in countries with warm climates such as Spain, a high prevalence of vitamin D deficiency has been identified, with 37.2% and 26.5% of the population having 25(OH)D values below 20 ng/mL during the winter and summer months, respectively [29]. Vitamin D deficiency is associated with an altered [19] lipid profile. According to Jiang et al. (2019) [30], low levels of 25(OH)D are associated with higher levels of LDL cholesterol (LDL-c) and triglycerides and lower levels of high-density lipoprotein cholesterol (HDL-c). Wang et al. (2016) [31] found that associations between serum 25(OH)D levels and serum lipids were more pronounced in men than in women, and higher incidences of elevated triglycerides and reduced HDL-cholesterol were associated with lower 25(OH)D levels in men.

Monocytes are immune cells involved in the release of various pro-inflammatory and pro-oxidants cytokines as part of the immune response. However, an increased monocyte count is associated with subclinical inflammation and the development of atherosclerosis [32]. Conversely, HDL-cholesterol exerts anti-inflammatory effects and down-regulates monocyte activation (Murphy et al., 2008 [33]). Currently, the ratio of monocytes to HDL-cholesterol (MHR) represents a novel biomarker of subclinical inflammation, as it reflects the balance between inflammatory and anti-inflammatory factors [34]. Kanbay et al. (2014) [35] first observed that MHR was a predictor of cardiovascular events in patients with chronic kidney disease (CKD). Subsequently, MHR has been proposed as a predictor of severity of coronary artery disease, peripheral artery disease, renal function and metabolic syndrome, among other conditions [36,37,38,39].

Some authors have explained that interactions between the immune system and the microbiome are linked to diet and that malnutrition in Western countries contributes to a state of chronic inflammation. Since diet could be an easily malleable factor, diet-induced changes and metabolism-related pathologies are highly dependent on food intake. The gut microbiota represents an indicative factor denoting adherence or non-adherence to a healthy diet, such as the Mediterranean diet. This type of dietary pattern is also associated with greater diversity and better gut barrier function and permeability than those observed in the Western pattern [40,41]. Nutrition education interventions among university students have been shown to be useful in promoting healthy eating [42,43]. However, there is little evidence on the long-term impact of these dietary improvements on different cardiometabolic health biomarkers in this population. Therefore, the aim of this study was to evaluate the effect of six months of a nutrition course on cardiometabolic parameters and food consumption frequency in undergraduate health science students.

## 2. Materials and Methods

### 2.1. Study Design and Participants

A quasi-experimental design was conducted with data collection before and after the nutrition education course was completed in a non-randomised university sample. The sample size was estimated at 40 participants with G *Power software (3.1.9.7; Heinrich Heine University of Düsseldorf, Düsseldorf, Germany), applying a probability of error α of 0.05. Participants were Spanish university health sciences students from a private university in Madrid. After convenience sampling, the aim of the study was explained to the students, and informed consent to be included in the study was signed. Assessment protocols were physically provided; data were analysed anonymously and loaded into a database for analysis.

**Figure 1:**
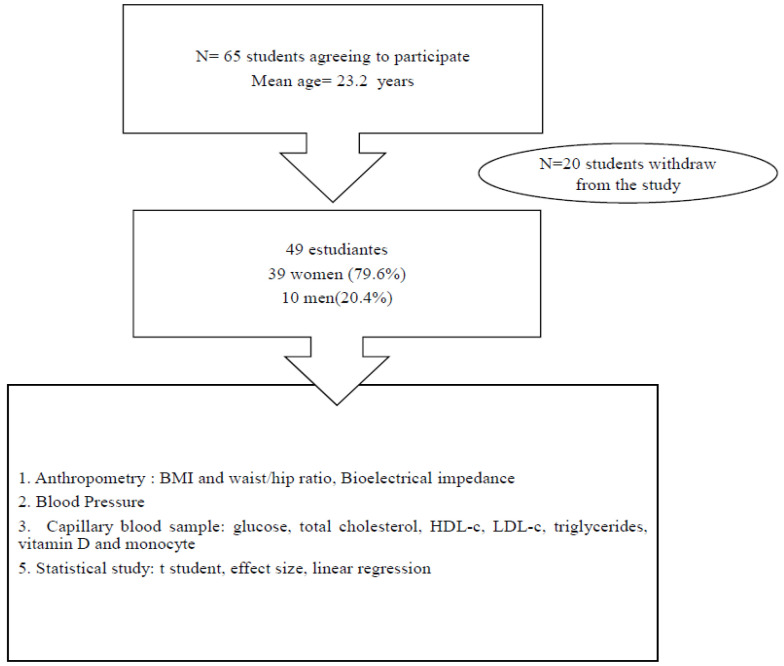
Flow chart of the study.

The study started in the academic year 2021–22. From September to December 2021, the students attended anatomy and physiology classes (prior to nutrition education). Data were collected in September 2021, and 69 undergraduate students were initially enrolled in the study. From January 2022 to June 2022, students completed their nutrition–physiopathology education. Finally, 49 undergraduate students attended all nutrition education sessions. Inclusion criteria were healthy students without comorbidities and exclusion criteria were students older than 25 years; students with missing data; students supplementing with vitamin D; pregnancy and comorbidities such as cardiovascular diseases, hypothyroidism or diabetes; as well as low energy intake (Figure 1).

The study protocol and design were approved by the Ethics Committee of the University Francisco de Vitoria (19/2022) and fully complied with the 1964 Helsinki Declaration and its subsequent amendments.

### 2.2. Description of Nutrition Education

Undergraduate students received physiology classes from September to December 2021. During the second part of academic year, they were taught nutrition and physiopathology from January 2022 to June 2022. Throughout this period, they received information during one and a half hour sessions twice a week (3 h per week) in nutrition, within a nutrition education curriculum (Table 1).

### 2.3. Analytical Procedure

The dietary habits of the participants were assessed using the Food Frequency Questionnaire (FFQ) before and after the course. We used a FFQ at the beginning and at the end of the course based on dietary habits in the last month. Food group categories were used: servings of fruit, vegetables, cereals and dairy/day and servings of fish, nuts, eggs, legumes and meat/week. To facilitate the correct interpretation of the FFQ, each food group was accompanied by a table of equivalence of servings according to previous studies [44]. The results obtained were compared with Spanish sustainable dietary recommendations [45]. Blood pressure (BP), including both systolic blood pressure (SBP) and diastolic blood pressure (DBP), was measured by a trained nurse using a standard technique to categorise students with optimal BP (SBP/DBP) (<120/80 mmHg), normal/high (120/80–140/90 mmHg) and elevated (≥140/90 mmHg) blood pressure. Capillary blood samples were collected at the same time from students after the overnight fasting period. Fasting blood glucose (FBG), serum total cholesterol, LDL-cholesterol, HDL-cholesterol, triglycerides, transferrin and ferritin were measured with a POC multiparametric luxmeter (Biochemical Systems International, Madrid, Spain). Serum 25-OH vitamin D was measured via immunochromatography with a Microcaya analyser (Microcaya, BilbaoCity, Spain). Blood analyses were completed with the HemoCue^®^ WBC monocyte count system (Madrid, Spain). The atherogenic index was calculated as a logarithm of triglycerides to HDL-cholesterol [46].

### 2.4. Anthropometric Measurements

Anthropometric measurements were recorded at baseline and after the course following standardized procedures, using calibrated digital scales SECA^®^ (SECA Vogel & Halke, Hamburg, Germany) as well as portable stadiometers SECA^®^ (SECA Vogel & Halke, Hamburg, Germany). Body weight measurements were taken barefoot and in light clothing, in kilograms, with an accuracy of 100 g unit (0.1 kg). Height was measured with the subject standing fully erect, feet together, head in the Frankfort plane and arms hanging freely, with an accuracy 0.1 cm. Body mass index (BMI) was calculated using the formula weight (kg)/[height (m)^2^]. Subjects were classified as underweight (BMI < 18.5 kg/m^2^), normal weight (BMI 18.5–24.9 kg/m^2^), overweight (BMI 25–29.9 kg/m^2^) and obese (BMI ≥ 30 kg/m^2^) [38,39,40]. Bioelectrical impedance analysis (BIA) was used to determine total body water, fat mass and fat-free mass in the students, with an InBody S10 (Inbody Co., Ltd., Seoul, Republic of Korea). Waist circumference was measured using an anthropometric tape measure in a horizontal plane midway between the lowest rib and the iliac crest. A waist-to-hip ratio >0.85 for females and >0.9 for males was used as a cut-off point for the identification of metabolic disease risk. The waist–hip ratio was also calculated, using the following formula: circumference of the waist (cm)/hip circumference (cm) [47].

### 2.5. Statistical Analysis

The continuous variables were expressed as mean and standard deviation (SD), while categorical variables were expressed as percentages and frequencies. Normality was confirmed using a Shapiro–Wilk test. A Student’s *t*-test was performed to analyse the relation between quantitative variables and categorical variables with two levels. One-way ANOVA was used to compare quantitative variables and categorical variables with more than 2 levels. The effect size was assessed with Cohen’s d, and the following benchmarks were established: small (d = 0.2), medium (d = 0.5) and large (d = 0.8) [48]. Linear regression analyses adjusted for gender, age and sex were performed when there was a significant association between the variables. The level of statistical significance was set at *p* < 0.05. The statistical analysis was performed using the IBM Statistical Package for Social Sciences (SPSS) version 22.0 (IBM, Chicago, IL, USA).

## 3. Results

At the end of the course, 49 students completed the study: 10 men (20.4%) and 39 women (79.6%); the sex ratio was 0.26. Regarding anthropometric variables, 71.4% of students had a BMI classified as normal weight (18.6–24.9 kg/m^2^) and 28.6% of participants were overweight (BMI 25–29.9 kg/m^2^). Also, men had higher W/H values (t = 3.5, d = 1, *p* = 0.001) and lower levels of fat mass (t = −7.1, d = 1.7, *p* < 0.05) compared to women. In terms of BP, significant gender differences were found, as both SBP (t = 2.4, d = 0.9, *p* = 0.019) and DBP (t = 2.3, d = 1.1, *p* = 0.044) were higher in men. Age, anthropometric characteristics and BP are shown in Table 2.

Table 3 shows the changes in food consumption frequency resulting from the course and alignment with dietary recommendations. After the course, a significant increase in the consumption of vegetables, fish, nuts and pulses was identified (*p* < 0.05). Similarly, there was a significant decrease in meat consumption (*p* < 0.05).

After the nutritional course, no significant changes were observed in BMI (22.9 pre-course vs. 22.4 post-course; *p* > 0.05), waist/hip ratio (0.8 pre-course vs. 0.8 post-course; *p* > 0.05), fat mass (23.5% pre-course vs. 26.7% post-course; *p* > 0.05), SBP (111 mmHg vs. 109.8 mmHg; *p* > 0.05) or DBP (65.4 mm Hg vs. 63.6 mm Hg; *p* > 0.05). Table 4 shows the effect of the nutrition education course on the metabolic profile. FBG levels were significantly decreased (t = −2.09, d = 0.30, *p* = 0.042), although the change in FBG occurred only in males (Δ = −10.9 mg/dL, *p* = 0.012) and not in females (Δ = −2.09, *p* = 0.322). Likewise, changes in the lipid profile were also found. HDL-cholesterol concentration increased significantly after the nutrition education course (t = 3.84, d = 0.55, *p* < 0.001), while in the case of LDL-cholesterol levels, a significant reduction was observed (t = −2.13, d = 0.54, *p* = 0.038). When stratified by gender, women showed an increase in HDL-cholesterol levels (Δ = 5.64, *p* = 0.007), but a significant decrease in LDL-cholesterol levels was not found (Δ = −6.53, *p* = 0.189). On the other hand, males exhibited a significant increase in HDL-cholesterol (Δ = 8.10, *p* = 0.001) and a strong decrease in LDL-c (Δ = −19.8, *p* = 0.043). The atherogenic index was also significantly reduced (t = −4.24, d = 0.57, *p* < 0.001) in both males (Δ = −0.55, *p* = 0.009) and females (Δ = −0.36, *p* = 0.002). No relationship was reported between the biomarkers analysed and changes in the intake of specific food groups, except for pulses. Legume consumption was associated with a reduction in LDL-c (F = 4.95, *p =* 0.002).

A significant increase in vitamin D levels was reported after the nutrition education course (t = 4.49, d = 0.65, *p* < 0.001). When classified by gender, the increase was greater in men (Δ = 7.94, *p* = 0.016 in men vs. Δ = 4.96, *p* = 0.001 in women). The WHR showed a significant reduction (t = −2.10, d = 0.27, *p* < 0.042), although these differences were only significant in men (Δ = −0.92, *p* = 0.01).

Table 5 shows the fat mass and lipid metabolism as a function of serum vitamin D concentrations after the course. Participants with adequate vitamin D levels (≧30 ng/mL) showed lower amounts of fat mass compared to those with inadequate vitamin D levels (30 ng/mL) (t = 3.02, d = 0.88, *p* = 0.004). In the case of HDL-cholesterol, a higher concentration was found in those participants with adequate vitamin D levels (t = −2.31, d = 0.67, *p* = 0.025). On the other hand, students with inadequate vitamin D levels had higher values of LDL-cholesterol (t = 2.58, d = 0.68, *p* = 0.01) and atherogenic index (t = 2.26, d = 0.61, *p* = 0.029). No differences were observed in total cholesterol and triglycerides levels.

From the different adjusted regression analyses, significant differences were only found for vitamin D and HDL-c and for vitamin D and MHR. Adjusted linear regression analysis showed a significant association between post-course vitamin D and post-course HDL-c (β = 0.40, IC: 0.18, 0.98, *p* < 0.01). Similarly, there was also a significant inverse relation between post-course vitamin D and MHR (β = −0.42, IC: −0.29, −0.06, *p* < 0.01) [19].

## 4. Discussion

In line with the original hypothesis, the results of the present study suggest that the acquisition of nutritional knowledge following six months of a nutrition education program can lead to a significant improvement in glycaemic metabolism, lipid profile, vitamin D levels and MHR.

Limited knowledge of nutrition and its relation to health has been observed among young people, underlining the need for nutrition education [49]. Food intake during the pandemic was reported to increase due to emotional eating as a means of comfort and to feel better in response to states of anxiety [15,17,18,19], with higher levels of this behaviour reported in women. Unhealthy lifestyle behaviours were also found to be positively associated with symptoms of depression and anxiety. Thus, understanding the lifestyle behaviours of university students during COVID-19 will help public health authorities to reshape future policies on their nutritional recommendations, in preparation for future pandemics [50,51]. López-Moreno et al. (2020) investigated the importance of adopting a healthy lifestyle for the duration of the COVID-19 pandemic. Similarly, future studies should assess whether these changes have been maintained after the pandemic situation [52]. Several studies have demonstrated the effectiveness of nutrition education interventions in university students [42,53]. The Special Turku Coronary Risk Factor Intervention Project (STRIP) evaluated the effect of a dietary intervention on different risk factors for atherosclerosis during childhood up to the age of 20 years. Pahkala et al. (2020) [54] conducted a 6-year post-intervention follow-up and found a higher adequacy of LDL-cholesterol levels, FBG and homeostatic model assessment of insulin resistance (HOMA-IR) in STRIPE participants compared to the control group. Similarly, we also found a decrease in FBG levels and LDL-cholesterol and an increase in HDL-cholesterol after the course. This could be due to a shift towards a healthy dietary pattern, as nutritional knowledge has been reported to correlate negatively with the consumption of unhealthy fats and cholesterol in the university population [55]. The importance of these results lies in the need to maintain optimal LDL-cholesterol levels from an early age considering that cardiovascular risk depends on the time course of LDL-cholesterol exposure [56].

In the present study, a higher consumption of pulses was associated with a reduction in LDL-cholesterol after the nutritional course. Several studies have demonstrated the benefits of legumes consumption on different cardiovascular risk factors, such as LDL-cholesterol [57]. In this regard, it has been reported that this significant reduction in LDL-cholesterol is achieved with a legume intake of 1 serving/day [58], similar to the consumption data reported after the nutritional course. Similarly, replacing red meat with high quality protein sources (legumes, soy and nuts) leads to a reduction in total and LDL-cholesterol [59]. In our study we did not specifically assess red meat consumption, but we did observe a reduction in meat consumption, which together with the increase in legume intake, could reinforce the observed effects. These beneficial effects of pulses could be due to their content of fibre, phytate and bioactive compounds such as polyphenols and saponins [60,61,62]. Dietary changes observed in the other foods were not associated with improvements in the biomarkers analysed. This suggests that the change in dietary pattern, rather than specific food groups, was the trigger for improvements in cardiometabolic profile and inflammatory status.

Body weight reduction is one of the most effective strategies to improve various metabolic parameters [63]. However, no anthropometric changes were observed in the present study, suggesting that the observed changes in the metabolic profile may be due to lifestyle improvements beyond changes in body weight. In the original STRIP study, an improvement in different parameters of the metabolic syndrome was observed in boys at the end of the course despite no change in waist circumference [64].

Poor-quality diet, physical inactivity, disruption of rest or stress can trigger chronic low-grade inflammation, characterized by persistent and sustained production of pro-inflammatory factors [65]. Numerous studies have shown that chronic low-grade inflammation is an important risk factor for chronic non-communicable diseases such as obesity, diabetes and cardiovascular disease [66,67]. C-reactive protein (CRP) is one of the traditionally used biomarkers of chronic inflammation [68]. In recent years, MHR has been proposed as a new marker of inflammation and oxidative stress [69]. In this regard, in our work, the nutritional education course led to a significant reduction in the MHR used as a marker of chronic inflammation. Previous studies have shown that in obese subjects an improvement in nutritional knowledge correlates with a decrease in CRP [70]. So far, no studies have evaluated the impact of diet on MHR; however, increased consumption of plant-based foods has been associated with a reduction in inflammation markers such as high-sensitivity C-reactive protein (hs-CRP), interleukin 1 beta (IL-1β), interleukin 6 (IL-6), Tumor necrosis factor-α (TNF-α) and transforming growth factor-beta (TGF-β) [71]. In the present study, the nutritional education course led to an improvement in dietary knowledge and, in principle, diet quality, which justifies the improvement in inflammatory status observed in previous studies that have reported how increased vegetable consumption reduces different markers of inflammation [72,73]. These results are highly relevant considering that MHR acts as an independent predictor of cardiovascular and all-cause mortality in the general population (Jiang et al., 2022 [34]).

Currently, a high prevalence of vitamin D deficiency has been detected worldwide [27,74]. Akimbekov et al. (2020) pointed that the interaction between VDR and Vitamin D directly influences the composition of the gut microbiota and reduces the release of pro-inflammatory cytokines. Thus, an inadequate level may impair normal gut homeostasis and barrier functions [75].

The university population showed a lack of awareness of the importance of vitamin D in bone health and other health-related physiological actions [76,77,78]. In the present study, we observed that vitamin D levels increased significantly after nutrition education. These findings are highly relevant considering the low vitamin D levels at baseline and their potential health implications. Goodman et al. (2016) [79] conducted an intervention in young adults (18–25 years) with the aim of improving vitamin D knowledge that led to an increase in dietary vitamin D intake. In this study, no changes in vitamin D blood levels were found, which may be partly due to the multiple environmental factors involved in vitamin D concentration, such as exercise, seasonal sun exposure or calcium intake and the limited duration of the intervention (12 weeks). In addition, it is important to note that the amount of dietary vitamin D required to increase plasma levels differs markedly among individuals [80].

Furthermore, among students with inadequate vitamin D levels, an increase in fat mass and a worsening of the lipid profile were observed. Some studies reported that vitamin D deficiency was positively associated with obesity mainly in abdominal fat [81]. In obese subjects, a 10-fold higher concentration of vitamin D has been found in adipose tissue compared to the plasma level [82]. This suggests that vitamin D, being a fat-soluble vitamin, is sequestered in adipose tissue, which decreases its bioavailability [71,73,74,75,76,77,80,83,84]. In addition, excess adiposity causes a down-regulation in the expression of cytochrome P450 2R1, the enzyme responsible for catalysing the formation of 25-hydrovitamin D from vitamin D [85]. Therefore, the lower synthesis of this enzyme reported in obese subjects may contribute to lower levels of circulating vitamin D.

Similar to our results, numerous studies have found an association between vitamin D deficiency and dyslipidaemia [86,87,88,89]. In our work, we observed that participants with inadequate plasma vitamin D levels had higher LDL-cholesterol, similar to previous studies [90,91]. Khayyatzadeh et al. (2018) [92] found that vitamin D supplementation in vitamin D-deficient adolescents resulted in a further reduction in LDL-c. Vitamin D receptors (VDR) have been identified in adipocytes, indicating the implications of vitamin D in the regulation of adipose tissue. In particular, vitamin D appears to exert an anti-adipogenic effect through multiple mechanisms, including inhibition of the synthesis of transcription factors involved in adipocyte differentiation and induction of apoptosis of mature pre-adipocytes [83]. Similarly, vitamin D, through its binding to the VDR, is also involved in the regulation of plasma cholesterol [93]. Lee et al. (2013) [94] showed that total cholesterol and oxidized LDL-cholesterol levels were inversely associated with serum vitamin D in children with obesity.

Another significant finding in this study was the inverse association between post-course vitamin D and MHR. Low vitamin D levels have been linked to an increase in chronic low-grade inflammation through its direct action on different pro-inflammatory cytokines and chemokines released by macrophages [95]. Previous studies have reported that low levels of vitamin D correlate with increased MHR, independent of metabolic and age status [96,97]. These findings could be due to several mechanisms. Vitamin D is involved in the down-regulation of monocyte adhesion molecules such as PSGL-1, β1-integrin and β2-integrin and in the activation of the anti-atherogenic monocyte/macrophage phenotype [98]. In particular, vitamin D inhibits M1 macrophage activation and promotes M1-M2 phenotype switching [99]. Moreover, monocyte count is a clinical parameter that increases in inflammation status linked to HDL-cholesterol depletion [100]. Therefore, vitamin D improves the inflammatory response, due to its anti-inflammatory effect on monocytes and pro-inflammatory cytokines [101]. Moreover, vitamin D plays an important role in lipoprotein metabolism and in very-low-density lipoprotein (VLDL-cholesterol) receptor expression, which promotes an increase in HDL-cholesterol [102]. Similarly, HDL-cholesterol acts as a protective factor in inflammation and antithrombotic and antioxidant effects by inhibiting monocyte recruitment to the arterial wall and enhancing vasorelaxation [103,104].

It is important to note that our study has some limitations. One of the main limitations was that dietary intake was not quantified, which makes it difficult to know the contribution of each macronutrient to the total diet and their association with the changes observed in cardiometabolic and inflammatory markers. Another limitation was that we performed a pilot study, so this study is limited by its sample size (N = 49), and the disproportion between men and women, which could limit the representativeness of the study population. However, this phenomenon has been reported in previous studies, since women represent a higher proportion in university health sciences students [10,105,106]. Another limitation of the study is that education was provided, but no knowledge results were reported. The change in food group intakes does not reflect knowledge, but food intakes.

## 5. Conclusions

In the university population, interventions aimed at improving nutritional knowledge and dietary habits have the potential to induce beneficial effects on various cardiometabolic markers and immune status. The course led to changes in dietary patterns, with an increase in the consumption of vegetables, nuts and legumes. In this study we observed an improvement in FBG, lipid profile, vitamin D levels and the MHR after the nutrition education. Similarly, an association was also observed between low vitamin D levels and MHR, used as a biomarker of chronic low-grade inflammation. These findings suggest the importance of including nutrition education programs during the university stage for the prevention of long-term health problems. To this end, more nutritional counselling and dietary strategies should be implemented in all Spanish Universities’ curricula to prevent or improve dietary habits in university students, which could help prevent different comorbidities in their lives. The aim should be to ensure the maintenance of the Mediterranean dietary pattern and to prevent deficiencies in the following generations.

These strategies are of great interest for public health considering their cost-effectiveness and affordability.

## Figures and Tables

**Table 1:** Topics and objectives of sessions during the nutrition course.

Topics	Session Objectives
Macronutrients	Understand the importance of carbohydrates, fibre, proteins, and lipids: classification, metabolism, and physiological function. Develop the skills necessary to optimise body composition.
Vitamins, minerals, and water	Understand the importance of micronutrients, dietary recommendations to achieve the total daily intake to prevent the risk of illness or injury. Understand the hydration–dehydration binomial and the role of minerals in the optimal functioning of the immune system.
Bioactive compounds, Mediterranean diet	Understand the role of polyphenols and human health: the role of bioavailability. Understand the role of the Mediterranean diet as an antioxidant protector. Extra virgin olive oil: dietary and health importance.
Energy balance model, body composition, dietary patterns and energy expenditure	Understand the energy balance model.
Nutrition during pregnancy, breastfeeding, childhood, adolescence, menopause, and old age	Understand the nutritional requirements throughout the life-periods and develop the skills necessary to improve nutrition during these periods of life.
Sports nutrition	Understand the importance of nutrients during exercise, the nutrition plan in the training–competition period and hydration–rehydration.
Diet in pathologies	Understand the physiopathology of different diseases and dietary prevention to improve the health of the population.
Obesity	Understand that obesity is a state of pathological increase in the amount of adipose tissue, which increases the risk of many diseases, such as cardiovascular disease, some cancers and type 2 diabetes.
Nutrigenetic and nutrigenomic	Understand how nutrigenomics studies and how environmental factors, such as food intake and lifestyle, influence genome expression.
Microbiota	Understand that the microbiota is essential for gastrointestinal function, as it is involved in the synthesis of vitamins, digestion and metabolism of carbohydrates and other dietary components, as well as in the development and function of the gastrointestinal immune system.

**Table 2:** Descriptive characteristics of the study sample by gender.

	All (n = 49)	Men (n = 10)	Women (n = 39)	*p*-Value, ES
Age (years)	23.2 (6.6)	23.6 (SD 7.5)	23.1 (SD 6.4)	ns, 0.1
Height (m)	1.66 (0.08)	1.78 (SD 0.09)	1.64 (SD 0.06)	<0.01, 2.1
Weight (kg)	63.9 (SD 10.6)	74.4 (SD 9.9)	61.2 (SD 9.1)	<0.01, 1.4
BMI (kg/m^2^)	22.9 (SD 2.9)	23.6 (SD 2.4)	22.7 (SD 3.1)	ns, 0.2
Waist (cm)	76.6 (SD 9.9)	81.5 (SD 8.6)	75.3 (SD 10.0)	ns, 0.6
Hip (cm)	97.5 (SD 10.5)	94.0 (SD 8.7)	98.4 (SD 10.8)	ns, 0.4
W/H	0.8 (SD 0.1)	0.9 (SD 0.1)	0.8 (SD 0.1)	<0.01, 1.0
Fat mass (%)	23.5 (SD 8.2)	14.3 (SD 3.6)	25.9 (SD 7.3)	<0.05, 1.7
Fat free mass (kg)	48.2 (SD 8.9)	63.6 (SD 7.7)	44.3 (SD 3.2)	<0.01, 5.0
SBP (mm Hg)	111.5 (11.4)	119.0 (8.8)	109.6 (11.3)	<0.05, 0.9
DBP (mm Hg)	65.4 (SD 7.9)	71.7 (SD 10.6)	63.7 (SD 6.2)	<0.05, 1.1

Data are presented as mean (standard deviation). BMI (Body Mass Index), W/H (Waist/Hip ratio), SBP (Systolic blood pressure), DBP (Diastolic blood pressure), ES (Effect size). Student *t*-test was conducted for continuous measurements, ns (no significative).

**Table 3:** Effect of the course on the frequency of food consumption.

	Recommendation	Pre-Course	Post-Course
Fruits	5 servings/day	0.8 servings/day	1.2 servings/day
Vegetables		2.6 servings/day	3.5 servings/day *
Cereals	3–6 servings/day	4.8 serving/day	4.6 serving/day
Dairy	0–3 servings/day	2.2 serving/day	2.1 serving/day
Fish	≥3 servings/week	3.9 servings/week	3.5 servings/week *
Nuts	≥3 servings/week	2.2 servings/week	3.9 servings/week *
Eggs	0–4 servings/week	3.6 servings/week	3.4 servings/week
Legumes	4–7 servings/week	1.2 servings/week	5.1 servings/week *
Meat	0–3 servings/week	5.6 servings/week	2.6 servings/week *

Data are presented as servings/day or servings/week in each of the food groups. Recommendations correspond to the Spanish guidelines for a sustainable diet [45]. Student *t*-test was conducted for continuous measurements. * Indicates significant differences between pre-course and post-course consumption.

**Table 4:** Effect of the course on biomarkers.

	Reference Values	Pre-Course	Post-Course	Post-Pre Changes	*p*-Value, ES
FBG (mg/dL)	70–110	84.3 (12.8)	80 (6.8)	−3.9 (13.0)	<0.05, 0.30
Cholesterol (mg/dL)	115–200	171.9 (32.3)	167.4 (29.9)	4.5 (33.6)	ns, 0.13
HDL-cholesterol (mg/dL)	35–75	58.7 (10.9)	64.9 (11.8)	6.1 (11.2)	<0.01, 0.55
LDL-cholesterol (mg/dL)	<150	97.8 (28.0)	88.5 (28.1)	−9.3 (30.3)	<0.05, 0.54
TG (mg/dL)	20–200	77.0 (35.1)	79.2 (31.9)	2.2 (40.8)	ns, 0.05
Atherogenic index	<4.5	3.0 (0.8)	2.6 (0.7)	0.4 (0.7)	<0.01, 0.57
Iron (µg/dL)	27–145	70.9 (18.2)	75.2 (17.2)	4.2 (25.3)	ns, 0.16
Transferrin (mg/dL)	175–400	316.6 (51.1)	316.0 (48.5)	−0.6 (44.2)	ns, 0.01
Ferritin (ng/mL)	15–150	52.9 (39.9)	54.5 (52.0)	1.6 (50.9)	ns, 0.30
Vitamin D (ng/mL)	≥30	22.6 (7.9)	28.2 (8.0)	5.6 (8.6)	<0.01, 0.65
MHR		8.9 (3.3)	7.9 (2.6)	0.9 (3.3)	<0.05, 0.27

Data are presented as mean (standard deviation). FBG (Fasting blood glucose), HDL-cholesterol (High-density lipoprotein-cholesterol), LDL-cholesterol (Low-density lipoprotein-cholesterol), TG (Triglycerides), MHR (Monocyte-to high density lipoprotein ratio), ns (not significant), ES (Effect size). Paired samples *t*-test to explore the differences in pre- and post-course.

**Table 5:** Differences in lipid metabolism indicators between those with insufficient and sufficient post-course vitamin D levels.

	Inadequate Vitamin D (<30 ng/mL) (n = 28)	Adequate Vitamin D (≥30 ng/mL) (n = 21)	*p*-Value, ES
Fat mass (kg)	17.4 (6.4)	11.4 (7.4)	<0.01, 0.88
Cholesterol (mg/dL)	169.8 (34.5)	164.2 (22.9)	ns, 0.19
HDL-c (mg/dL)	61.6 (9.7)	69.1 (13.1)	<0.05, 0.67
LDL-c (mg/dL)	96.4 (32.6)	78.1 (16.1)	<0.05, 0.68
TG (mg/dL)	74.9 (27.2)	85.0 (37.3)	ns, 0.32
Atherogenic index	2.8 (0.8)	2.4 (0.4)	<0.05, 0.61

Data are presented as mean (standard deviation). HDL-cholesterol (High-density lipoprotein-cholesterol), LDL-cholesterol (Low-density lipoprotein-cholesterol), TG (Triglycerides), ns (not significant), ES (Effect size). Student *t*-test was conducted for continuous measurements.

## Data Availability

The datasets generated during the current study are publicly available.
